# Implemented to last? Schools’ strategies for promoting the sustainability of the ‘60 minutes a day of physical activity’ initiative in Quebec primary schools

**DOI:** 10.1177/17579759241303488

**Published:** 2025-12-15

**Authors:** Suzanne Laberge, Véronique Gosselin

**Affiliations:** School of Kinesiology and Physical Activity Sciences, Faculty of Medicine, Université de Montréal, Canada.

**Keywords:** schools settings, daily physical activity promotion, sustainability strategies

## Abstract

**Background:**

Many studies have investigated the adoption and implementation of school-based physical activity interventions, but only a few have reported on their sustainability. Understanding the factors contributing to school-based physical activity interventions being maintained or abandoned is necessary to ensure that resource investments are effective.

**Objective:**

This study aims to contribute to this understanding. We investigated the actions and strategies developed by the school teams participating in the Quebec *Active at school!* initiative in ensuring that the tailored interventions they had developed over the three-year degressive funding were maintained.

**Methods:**

Target population was the 415 schools that reached the end of the three-year funding period in 2020. An online survey consisting of 27 multiple choice questions, each followed by open-ended questions in which respondents explained or justified their choice, was sent to all participating schools.

**Results:**

A total of 397 of the 415 schools responded to the survey. The analysis showed that the school teams have integrated actions to foster sustainability during initial project planning and adoption. Eight main strategies were deployed to sustain the interventions: maintenance of the most popular physical activities, inclusion of the 60-min daily physical activity (DPA) initiative in the school Educational Project, student involvement in implementing DPA, maintaining a person in charge of DPA implementation, maintaining the committee in charge of DPA implementation, training new staff, researching new sources of funding, maintaining developed collaborations.

**Conclusion:**

The initiative’s bottom-up approach has favored school teams developing various sustainability strategies, whether at the institutional, organizational or community level. Given the acceleration of contemporary changes, it is inevitable that DPA interventions will need to be adapted and transformed. Therefore, what should be sustained is the schools’ capacity building and innovativeness that has been generated by the new program implementation.

## Introduction

According to the World Health Organization (1), about 80% of school-aged children living in industrialized countries do not meet the physical activity recommendations of at least 60 min of moderate to vigorous physical activity daily. Given the various health benefits of physical activity (2), and the association that has been found between physical activity levels in youth and adulthood (3), promoting physical activity in school-aged children is a concern for public health. School settings are promising sites for interventions, especially because young people in most parts of the world spend a lot of time attending school (4). A wide variety of programs and initiatives promoting physical activity in schools have been launched in the last decades, many of them inspired by the comprehensive school physical activity framework (5). Yet one critical challenge of these programs has been the long-term viability of the interventions post-external funding. A recent systematic review examined the sustainability of multi-component school-based public health interventions and found that none of the interventions was entirely sustained once active implementation support (i.e. start-up funding and/or other resources) ceased (6). Of the 24 interventions studied, only eight targeted physical activity. Furthermore, many studies have investigated the adoption and implementation of school-based physical activity interventions, but only a few have reported on their sustainability (7). Understanding the factors contributing to school-based physical activity interventions being maintained or abandoned is necessary to ensure that resource investments are effective. This study aims to contribute to this understanding. We investigated the actions and strategies developed by the school teams participating in the Quebec *Active at School!* initiative in ensuring that the tailored interventions they had developed were maintained.

### The *Active at School!* initiative

In 2017, the Quebec government launched its first policy on sports, physical activity and leisure (8), which included legislative provisions mandating the integration of 60 min of daily physical activity (DPA) within all elementary schools. To support the schools, a specific program, Active at School!, was launched (9). This program provides financial resources to participating schools over three years, degressively, to implement opportunities for students to be active for 60 min each school day. The program’s underpinning goal was that allocating resources degressively and allowing the schools to develop a custom plan and implement actions tailored to their needs would foster a shift in the school culture towards a sustained provision of DPA. This logic reflects bottom-up, community-based approaches to policy-making, as the program’s implementation is based on the mobilization of a group (a school team), whose members come together to propose actions tailored to their context to initiate a change of practice (10). In theory, the school team is then more likely to ‘take ownership’ of the program, facilitating DPA sustainability and longer-term beneficial health impacts for the students. The program can be characterized as a top-down objective with a bottom-up implementation (11).

### Objectives

The aim of our study was to examine to what extent the school teams have adopted actions or strategies to foster the sustainability of the interventions they implemented over the three-year period of funded DPA initiative. More specifically, we sought to answer the following questions: given the specific context of local implementation, (1) what factors favored or hampered maintaining the commitment of the school team over the three-year period? (2) what strategies or actions did the school team plan in order to foster the sustainability of the interventions beyond the three years of financial support?

Ethical approval for the study was obtained from the Université de Montréal Multifaculty Ethics Board. Researchers had no access to names of the schools that answered the survey.

## Method

The research is a cross-sectional study analyzing data from an online survey.

### Participants

The participants were all of the schools (415) constituting the first cohort of the *Active at School!* program initiated in 2017. Schools participated on a voluntary basis. The resources allocated by the Ministry were to be used by the school teams to implement new practices, both at the level of the school organization (e.g. set up a committee, appoint an in-school leader, and so on) and the interventions themselves (e.g. schedule in-class active breaks or lead physical activities during recess). For the first year of implementation (2017), a maximum of 450 participating schools was set by the Ministry, and 415 joined the program. These 415 schools reached the end of the three-year funding period in 2020.

### Instrument

We drew on key elements of the sustainability framework proposed by Scheirer and Dearing (12), and the facilitating and hindering factors identified by the respective reviews of Herlitz *et al.* (6), Cassar *et al.* (7) and Zurynski *et al.* (13) to develop the survey questions. Items were selected for and adapted to the particular context of the program. The questions assessed the major factors likely to influence the sustainability of local interventions. An online survey consisting of 27 multiple choice questions, each followed by open-ended questions in which respondents explained or justified their choice, was sent to the people in charge of the implementation at all participating schools. A total of 397 schools responded to the survey (response rate of 96.3%). Although this is a convenience sample (14), this response rate is high enough to assume that the results are representative of the participating schools. Respondents included those in charge of the implementation of the initiative (mainly the school principal). We carried out descriptive statistics and thematic qualitative analyses of the collected data. The investigators analyzed separately the qualitative data to identify relevant categories for each question. Their respective sets of categories were then compared. Any differences in interpretation were resolved through discussion until consensus was obtained.

## Results

Because a physical activity-promoting program is more likely to be sustainable if the various members of the school team have at least maintained their commitment over the years funded, we first examined the evolution of the school team’s commitment and the factors that favored or hampered it. The results indicate that the commitment of the school teams to integrating active time into school daily life showed no signs of slowing down overall over the three years. In fact, respondents indicated that the different members of the school team significantly increased their involvement: 69.3% for classroom teachers, 61.5% for physical education teachers, 56.9% for daycare educators and 54.4% for special education teachers.

The analysis of the explanations provided by the respondents highlighted four main factors that facilitated the increase and three main factors hindering commitment. The facilitators were: evidence of leadership from the people in charge of the implementation, positive impacts observed on students, an increase in the students’ participation in the program activities, and improved recognition of the role of physical activity in educational success. The barriers were: staff turnover, educational priorities, and the need to adapt or update the interventions. Table 1 presents the frequencies of these factors and some quotations illustrating each one.

**Table 1.** Facilitators and barriers to maintaining commitment as reported by the respondents (frequency in %; *N* = 397).

Facilitators	*Quotes*
1. Evidence of leadership from the people in charge of the implementation	
1.1 Sharing information, useful activities and original initiatives (85.6%)	‘We used social media to share new means or new ways of doing things in order to offer students the best possible experience.’ ‘We organized conferences, shared articles on the subject, followed by discussions and sharing of ideas with the teacher in charge.’ ‘We organized training for staff on all the new equipment and did workshops with all the teachers. We prepared information packages with classroom activities for teachers.’
1.2 Identifying leaders or champions likely to contribute to maintaining mobilization (82.4%)	‘We have identified ‘champions,’ members of the school team who are respected, dynamic and who believe in the benefits of physical activity. These people are walking mobilizers for us.’
1.3 Establishing formal meetings to share successes and overcome challenges (65.3%)	‘The principal has added an item to the agenda of each monthly meeting in order to share information with the entire school team; also to share good things about the project.’
1.4 Publicly expressing recognition to members of the school team who have stood out through their involvement (48.1%)	‘We have organized a list of successes and new initiatives. Publicly expressing the contribution of people who stand out in their involvement is very important to keeping the school team mobilized.’
2. Positive impacts observed on students (82.4%)	‘Seeing the students being able to refocus after doing the activities was certainly an added bonus for the teachers. At first, it seemed like it would be an interruption, but they quickly noticed that it was worth the time once they began doing it. It was a win–win; for the teachers and the students.’ ‘Following the establishment of facilities for active travel, active corridors, active breaks, etc., several stakeholders shared their successes, and this gave rise to new initiatives. All this developed little by little and is still ongoing. People did not feel rushed and were happy to see the benefits in the students.’ ‘The activities helped reduce disciplinary problems for students with a great need to move. Several students also realized the benefits of trying new PA.’
3. Increase in the students’ participation in the program activities (65.0%)	‘Seeing the pleasure the students took in participating in our activities sent us a message that it was good, and it encouraged the school team to continue to involve everyone.’ ‘Over the three years, we have seen an increase in student participation in our activities, perhaps because we involved them in choosing and carrying out the activities. The school team had therefore embraced the mobilization!’
4. Improved recognition of the role of PA in educational success (48.1%)	‘People have become aware of the importance of PA. The discourse has changed. For example, take active breaks: before, teachers said that it annoyed the students. Now, the majority of teachers schedule one or more per day.’ ‘Many teachers now recognize that students need to move and that there is a positive impact on their concentration, and therefore on their educational success.’
Barriers	*Quotes*
1. Staff turnover (68.5%)	‘We had a change of management last year, so the leadership for maintaining our activities was not optimal.’ ‘Staff turnover among phys ed teachers and classroom teachers has slowed our activities. The new principal had to mobilize our leaders to continue activities regardless.’
2. Educational priorities (52.3%)	‘The DPA initiative coincided with the Educational Project to be accomplished, the implementation of sex education (the same year) and CAP (professional community of learning) as well as reading screenings (which had very low results). We tasked the daycare service with facilitating physical activities, because the teachers had to focus their energy on issues other than the DPA.’ ‘Many additions linked to the educational load required more motivation from staff as well as the need to make choices, and often, it was the time scheduled for movement [PA] which was lost.’
3. The need to adapt or update the interventions (38.2%)	‘Sometimes the time and adaptation required to set up and organize new activities creates pressure among classroom teachers and puts the maintenance of activities at risk.’

PA: physical activity; phys ed: physical education; DPA: daily physical activity

### Strategies implemented to foster the sustainability of the interventions

A strong majority of schools (78.3%) said they had put various strategies in place to maintain the commitment of their school team and the interventions at the end of the funding. Schools were invited to identify their strategies. The analysis of their responses revealed eight main strategies, presented in the following paragraphs, with quotations.

#### 1. Maintaining the most popular physical activities

**Figure 1.**
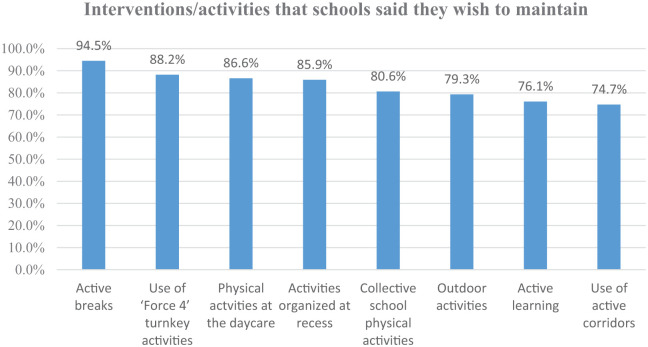
Percentage of schools that intend to maintain some of the activities developed.

Maintaining the activities developed over the three years appeared to be the main strategy to sustain the DPA initiative. Beyond the more school-specific and customized activities (more than 200 different organized activities were reported), some interventions proved popular in the majority of schools. These are indicated in Figure 1.

The active breaks implemented in the classroom appeared to be the easiest to set up and the most appreciated by the students, especially the younger ones. The activity videos provided by Force 4 (a non-profit organization promoting physical activity in schools) consist of physical activity routines with popular music; it has the advantage of requiring little preparation for the teachers and students can be involved in leading the routines.

#### 2. Including the 60-min DPA initiative in the school Educational Project

The educational project is a public document that defines the school orientations, action priorities and expected results to ensure the educational success of all students. It is designed in collaboration with different stakeholders of the school community: students, parents and staff of the establishment. In response to the question, ‘At the end of your participation in the initiative, did your school change the Educational Project to indicate that providing at least 60 minutes of DPA is one of your priorities?’, seven out of 10 (72.3%) schools indicated that they did. This statement document is crucial for sustaining the DPA interventions despite staff turnover.

‘The target of 60 minutes of DPA is part of our Educational Project. Through our committee, we offer a list of ways to increase the number of minutes of [physical activity] each day.’

‘In our Educational Project, we have a strategy directly linked to continuity. We want to increase students’ active time on a daily basis. Follow-up will be done with teachers regularly.’

‘To ensure the sustainability of an active lifestyle within a school establishment, it must be included in the Educational Project and shared by the school team and the community. That’s why we included it in the [Educational Project].’

#### 3. Student involvement in implementing DPA

About two out of three schools (66.5%) reported that they will continue to involve students, mainly the older ones, in the DPA provision.

‘We believe the involvement of the students is an asset for maintaining [physical activity] participation. It helps the teachers and facilitates the mobilization of their peers.’

‘We involved grade 6 students for organizing equipment that takes longer to install and also for making sure the schoolyard was functional for breaks. It gave them a sense of responsibility and it saved time for the teachers.’

#### 4. Maintaining a person in charge of DPA implementation

Nearly four out of five schools (78.6%) reported wanting to keep a person in charge of the DPA initiative.

‘We will keep a person in charge of the DPA initiative because this person will ensure the activities implemented are maintained and improved each year, and new staff are trained.’

‘Thanks to our teacher in charge of the DPA initiative, we implemented creative and innovative interventions and encouraged new developments at regular intervals (videos, capsules, meeting points with staff) to keep the developed interventions active and make them last over time.’

#### 5. Maintaining the committee in charge of DPA implementation

A lower percentage of schools (60.5%) said they were maintaining the committee established at the onset of the initiative.

‘The DPA committee will continue its activities. It will communicate information, suggest activities and outings, lead gatherings, etc. In addition, the committee’s mandate also consists of seeking partners and providing the team with opportunities to get students moving. Finally, the committee will continue to organize active corridors and animated recesses.’

#### 6. Training new personnel

Given the high staff turnover experienced in schools (34% among the physical education teachers and 20% among the class teachers) (15), maintaining interventions requires providing means to enable new staff to actively engage in providing active time to students. About three schools out of five (61.2%) reported that they have planned some strategies to train the new teachers.

‘There is a physical education teacher who is partially relieved of his teaching duties to ensure the implementation of the initiative in the school and to support the integration of new staff.’

#### 7. Researching new sources of funding

The financial resources required to offer certain physical activity, such as outdoor outings, are often cut to meet needs deemed to be priorities. If schools want to maintain them, they must consider fundraising strategies. This is what almost half of the schools (48.6%) did.

‘Given the end of the government subsidy, the school has set up a financing committee to raise nearly $30,000.00 annually to reduce costs to parents in organizing our sports component.’

‘Our Active at school committee has worked to identify funders for our outdoor outings. We are also considering creating a fund dedicated to maintaining activities that are more expensive but have proven effective in encouraging our students to be physically active.’

#### 8. Maintaining developed collaborations

**Figure 2.**
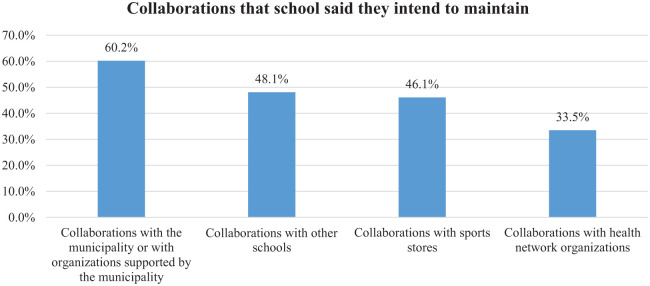
Percentage of schools that intend to maintain the developed collaborations.

The results suggest that the degressive nature of the government funding encouraged schools to develop collaborations with other schools or other organizations. Collaborations with municipalities have been the most promising and are prioritized (60.2%) as something to be maintained (Figure 2). Collaborations with the health network appeared less appealing for the school, presumably because of the significant difference in their management culture.

‘The excellent collaboration and partnership with the municipality for the grounds and sports equipment will promote continuation.’

‘Given the imminent end of funding, we have collected equipment (cross-country skis and snowshoes) from sports stores that can be used for physical activity. We also have agreements with the municipality giving us access to their facilities.’

## Discussion

This study aimed to examine to what extent the school teams participating in the 60-min DPA Quebec government initiative have adopted strategies to foster the sustainability of the interventions they planned and implemented over the three-year funded period. Two main findings emerged from the analyses. First, the school teams have integrated actions to foster sustainability during initial project planning and adoption. Second, the main strategies deployed to sustain the interventions were at three levels: institutional, organizational and community environment.

Concerning the first main finding, we have shown that increase in school teams’ commitment over the three years has been facilitated by actions boosting the schools’ capacity to maintain DPA interventions. Indeed, the establishment of formal meetings devoted to the initiative, the various ways for sharing DPA information and useful activities, and the identification of leaders were likely to prepare the school teams to be able to maintain the DPA interventions. This is consistent with the sustainability model proposed by Johnson *et al.* (16). The authors call this preparatory phase the ‘infrastructure capacity-building’ (16), which includes effective leadership, presence of champions, resources, et cetera. Moreover, the positive impacts of physical activity observed on students, the increase in the students’ participation in the program activities, as well as the improved recognition of the role of physical activity in educational success were favoring the reinforcement of the school team’s motivation to maintain the interventions after the three-year funding period. Studies (17,18) have shown that to maintain their involvement, teachers must find the interventions beneficial for students and advantageous for themselves. This can be linked to the reinforcement theory of motivation, according to which positive reinforcement with rewards reinforces desired behavior (19). Hence, the three-year period has allowed the school teams to build sustainability capacities and readiness (16).

Our second main finding is congruent with Pluye *et al.* (20)’s two social structures involved in sustainability: institutional and organizational structures. The inclusion of the 60-min DPA initiative in the Educational Project of the school and maintaining an appointed DPA coordinator represent changes at the institutional level. They are in themselves important outcomes of the initiative. Furthermore, they are sound sustainability strategies because they will have impacts at the lower level, that is, the organizational one. The latter can be related to three of the strategies reported by the participating schools: maintaining the most popular activities and their eventual adaptations, maintaining the committee in charge of DPA implementation and the means developed to ensure the training of the new personnel. The pivotal importance of the organizational aspect of program sustainability has already been highlighted by previous studies (17,18), literature reviews (6,7,13) and sustainability models (12,16,20). This aspect is usually the first to be considered by program planners; the participating schools of our study do not differ in this regard. Some authors even refer to the organizational aspect as ‘organizational routines’ (20), meaning that the interventions developed are stable and regular parts of the organizational procedures.

The third level (community environment) of the strategies reported by the schools to ensure the sustainability of their developed interventions concerns the maintenance of the developed collaborations and the search for new funding. The bottom-up nature of the DPA implementation may have favored collaboration with other schools in the surrounding environment (pooling resources and scaling the interventions) and with the local municipality (access to sports facilities). This suggests that the DPA initiative has led to changes not only within the school but within the surrounding community, thus creating a collaborative movement in favor of physical activity practice. A study on the sustainability of a student peer-led physical activity program in New Zealand (21) has also highlighted the positive and significant role of a collaborative approach in maintaining a program. Yet, it is argued that even if the initial intervention program is discontinued, maintaining community-level collaboration or partnership may stimulate new physical activity-promoting initiatives (12).

As for the search for new funding, our results indicated that only one school out of two has mentioned an action on this issue. Two hypotheses may explain the relatively low frequency: either their interventions did not need more financial resources, or this represents too many challenges and time investment. However, contrary to what is often thought, financial resources are not the essential determinant of maintaining a program. Scheirer and Dearing (12), rather, affirm that continued financial support is not synonymous with sustainability; the availability of resources should instead be considered as a significant influence on sustainability outcomes.

## Limitations

These findings enrich the understanding of the factors favoring the sustainability of a top-down/bottom-up school-based DPA initiative. However, our study has some limitations. First, the participating schools were self-selected to enroll in the program, limiting the generalizability of the findings. The participating schools may have been more motivated to engage in a physical activity-promoting initiative than non-participating schools. Second, the surveys provided self-reported data and may be subject to bias on behalf of schools. Finally, open-ended questions provide only short answers and do not allow more in-depth investigations. Nonetheless, using questionnaires to collect large amounts of qualitative and quantitative data allowed for both an overall and a detailed analysis of the strategies planned to ensure the sustainability of the physical activity-promoting initiative.

## Conclusion

With the *Active at School!* three-year degressive funding initiative, participating schools were in charge of planning and implementing DPA interventions tailored to their own context. Our study has observed that this type of approach has favored school teams developing various sustainability strategies, whether at the institutional, organizational or community level. Given the acceleration of contemporary changes, it is inevitable that DPA interventions will need to be constantly adapted and transformed. Therefore, as Green (22) suggested, what is more important to be sustained is the schools’ capacity building and innovativeness that has been generated by the new program implementation. Accordingly, it would be promising for policymakers to favor bottom-up approaches rather than top-down ones in promoting physical activity in the school setting, and to develop initiatives that support the schools’ capacity building and innovativeness rather than specific and unilateral interventions or actions. Future research is needed to investigate the capacity-building processes that are generated in the case of DPA bottom-up program implementation.
